# Effects of different regional cerebral blood flow on white matter hyperintensity in CADASIL patients

**DOI:** 10.7555/JBR.36.20220006

**Published:** 2022-08-28

**Authors:** Runrun Wang, Jiewen Zhang, Junkui Shang, Fengyu Wang, Xi Yan

**Affiliations:** Department of Neurology, Henan Provincial People's Hospital, Zhengzhou University People's Hospital, Henan University People's Hospital, Zhengzhou, Henan 450003, China

**Keywords:** cerebral hypoperfusion, neurovascular unit, white matter hyperintensity, small vessel disease, CADASIL

## Abstract

Cerebral autosomal dominant arteriopathy with subcortical infarcts and leukoencephalopathy (CADASIL) is an early-onset inherited small vessel disease. Decreased cerebral blood flow (CBF) may contribute to white matter hyperintensity (WMH) severity in CADASIL, but more evidence is needed to support this hypothesis. This study comprised six patients with CADASIL who harbored mutations in the coding sequence of *NOTCH3* and twelve age-matched neurologically healthy controls. We collected clinical and imaging data from patients with CADASIL and divided the brain into four regions: WMH, normal-appearing white matter (NAWM), gray matter (GM), and global brain. We analyzed the relationship between CBF of each region and the WMH volume. Compared with the control group, CBF was significantly decreased in all four regions in the CADASIL group. Lower CBF in these regions was correlated with higher WMH volume in CADASIL. CBF in the NAWM, GM and global regions was positively correlated with that in WMH region. However, after correction tests, only CBF in the WMH region but not in NAWM, GM and global regions was associated with WMH volume. Our findings suggest that CBF in the WMH region is an influencing factor of the WMH severity in CADASIL.

## Introduction

Cerebral autosomal dominant arteriopathy with subcortical infarcts and leukoencephalopathy (CADASIL) is the most prevalent hereditary cerebral small vessel disease (SVD) caused by mutations of the *NOTCH3* gene^[1]^. *NOTCH3* is a large gene with 33 exons that encodes a single-pass transmembrane receptor. It comprises a 210-kDa neurogenic locus notch homolog protein 3 (Notch3) extracellular domain (Notch3^ECD^) and 97-kDa Notch3 transmembrane intracellular domain (Notch3^TICD^)^[2]^. Typically, Notch3 is expressed in the vascular smooth muscle cells (VSMCs) and pericytes of small arteries in adults^[3]^. *NOTCH3* mutations result in Notch3^ECD^ aggregation in the media of small arteries, ultimately leading to vessel wall degeneration^[4]^. The pathological changes in small vessels include granular osmiophilic material (GOM) deposition surrounding VSMCs and pericytes, increased arterial wall thickness, arterial media fibrosis, and loss of VSMCs and pericytes^[5]^. Interestingly, both cerebral and extracerebral small arteries, such as skin vasculature, present these degenerative changes^[6–7]^. However, only cerebral small vessels have primarily compromised function in the early stage of CADASIL^[8]^, suggesting that cerebrovascular dysfunction may play an important role in the pathogenesis of CADASIL. Conversely, white matter hyperintensity (WMH) is the radiological hallmark of CADASIL^[9]^. Since cerebrovascular dysfunction precedes WMH^[10]^, it is presumed that WMH is of vascular origin and caused by cerebrovascular dysfunction, which makes it immensely significant to investigate the changes in cerebrovascular function in CADASIL.

Small vessels of the brain form an elaborate vascular network to help maintain normal brain functions^[11]^. The neurovascular unit (NVU) provides a useful framework to investigate the cerebral vascular network function^[12]^. VSMCs and pericytes, as the main cellular components of the NVU, play an important role in regulating NVU function^[13]^. The key function of the NVU is to regulate the cerebral blood flow (CBF) to nearby small vessels to support the metabolic needs of the local neuronal activity, which is called neurovascular coupling (NVC)^[12]^. NVU dysfunction manifests in different ways, including changes in cerebrovascular reactivity (CVR), intracranial vascular and cerebrospinal fluid (CSF) pulsatility, CBF, and integrity of the blood-brain barrier (BBB)^[14]^. Previous studies have evidenced NVU dysfunction in CADASIL, which presents as decreased regional cerebral blood volume^[15]^, lower CVR^[16]^, reduced CBF velocity^[17]^ and increased regional BBB permeability^[18]^. Reduced CBF velocity is also correlated with WMH severity^[17]^. These studies only focused on the cerebrovascular function in the WMH region.

Moreover, the deposition of Notch3^ECD^ and other extracellular matrix proteins around cerebral small vessels is not only found in the WMH region but also in other brain regions^[19]^. It is unclear whether the cerebrovascular function of other brain regions is also affected. Notably, decreased CBF could lead to endothelial cell activation, mural cell death, immune cell infiltration, microglia activation, and gliosis^[20]^. However, the cellular mechanisms that link cerebral hypoperfusion and WMH in CADASIL were not fully understood. Besides pronounced small vessel degeneration, which primarily presented as VSMC and pericyte losses, increased vessel wall thickness, lumen narrowing^[5]^, severe white matter astrocytopathy were also observed around the vessels^[21–22]^, suggesting severe impairment of the gliovascular unit after cerebral hypoperfusion in CADASIL.

Therefore, in this study, we first observed the changes of CBF in the WMH, normal-appearing white matter (NAWM), gray matter (GM), and global brain and recorded the WMH volume in CADASIL. Then, we investigated the relationship between changes in regional CBF and the WMH volume in CADASIL.

## Subjects and methods

### Patients and controls

In this study, six patients with CADASIL who harbored mutations in the coding sequence of *NOTCH3* (predicted to cause either the gain or loss of cysteine residues) and 12 healthy controls matched with these CADASIL patients in terms of age, gender, education level were recruited from the Henan Provincial People's Hospital. These normal controls had no neurological or psychiatric diseases or disease-associated harbor mutations in the coding sequence of *NOTCH3*, and their magnetic resonance imaging (MRI) did not meet the imaging criteria for cerebral small vessel disease. All enrolled participants provided written informed consent. The study design was approved by the Medical Ethical Committee of Henan Provincial People's Hospital (Approval No. 202077). At the time of enrollment, comprehensive physical examination, clinical interview, biochemical analysis, and brain MRI were performed for all patients.

### Brain magnetic resonance imaging

#### Imaging protocol

Brain MRI was performed using a Philips Achieva 3 Tesla system with an 8-channel head coil (Philips Healthcare, Netherlands). T2-weighted fluid-attenuated inversion recovery (FLAIR) images (with a resolution of 1 mm × 1 mm × 1 mm, inversion time [TI] of 1800 ms, echo time [TE] of 388 ms, and repetition time [TR] of 5000 ms) were acquired for WMH segmentation. Arterial spin labeling (ASL) was performed using a pseudo-continuous pulse sequence, and the set parameters were as follows: TR, 3400 ms; TE, 19 ms; TI, 2000 ms; FOV, 320×160; matrix, 64×32; slices, 26; voxel size, 5 mm × 5 mm × 4 mm; scan time, 4 min and 38 s. FLAIR parameters were as follows: TR, 9000 ms; TE, 150 ms; TI, 2250 ms; FOV, 24 cm × 24 cm; matrix size, 256×192; slice thickness, 5.0 mm. The T1-weighted structural image parameters were as follows: TR, 2400 ms; TE, 4 ms; slice thickness, 1.0 mm; voxel size, 1 mm × 1 mm × 1 mm.

#### Data processing

To obtain quantitative perfusion maps, the captured ASL images were processed using IDL 6.1 for Windows (ITT Visual Information Solutions, USA). First, head movement correction was performed; next, the baseline image and CBF were quantified on the basis of the Buxton hemodynamic model to obtain a specific formula for CBF quantification. Visual recognition and description of clinical information were blinded by a single trained rater.

We registered structural images of each participant. For each participant, we generated WMH probability maps using their FLAIR and T1W image data. In the experimental part, all the above segmentation algorithms were applied to two public datasets. We obtained final estimates using 3D smoothing where partial volume effects were accounted for and noise was reduced before manually removing indices and any prior stroke lesions. We segmented normal-appearing tissue and whole-brain volumes from each participant's T1W data and local population-specific probability maps. All tissue masks were visually inspected and manually corrected if necessary.

### Statistical analysis

The measurement data were expressed as mean±SD. Two independent samples *t*-tests were conducted to compare the continuous variables between the control and CADASIL groups. The Chi-square test was used to analyze the distributional difference of categorical variables. Spearman's rank correlation test and linear regression analyses were used to analyze the association between CBF in the different brain regions (WMH, NAWM, GM, and global brain) and the WMH volume. To detect the existence and severity of collinearity between CBF of different brain regions in the WMH volume predictive model, we calculated the linear dependence between CBF of different brain regions using Spearman's rank correlation test. In addition, the variance inflation factor (VIF) was used to assess the extent of variance inflation of the estimated coefficients. A predictor with VIF >10 was considered indicative of serious collinearity. Data analysis was performed using the the SPSS 23.0 software (IBM Corp., USA). Statistical significance was set at *P*<0.05 (two-tailed).

## Results

### Participant characteristics

A total of 12 neurologically healthy controls (as the control group, aged [45.67±10.58] years; male in 41.67%) and six patients with CADASIL who harbored mutations in the coding sequence of *NOTCH3* (as the CADASIL group, aged [52.00±16.11] years; male in 66.67%) were enrolled in this study. Participant characteristics are listed in ***Table 1***. The incidence of hypertension, diabetes, hyperlipidemia, and rate of smoking did not significantly differ between the two groups (*P*>0.05). There were no significant differences in systolic blood pressure, diastolic blood pressure, and levels of serum total cholesterol, triglycerides, high-density lipoprotein cholesterol, low-density lipoprotein cholesterol, and glucose (*P*>0.05) between the groups. However, the volume of WMH was significantly higher in the CADASIL group ([22.85±12.99] mL) than that in the control group ([0.39±0.36] mL) (*P=*0.008).

**Table 1:** Characteristics of the CADASIL and control groups

Characteristics	Control (*n*=12)	CADASIL (*n*=6)	*t/χ2*	*P*
Demographic data				
Male, *n* (%)	5 (41.67)	4 (66.67)		0.620^a^
Female, *n* (%)	7 (58.33)	2 (33.33)		
Age (years), mean±SD	45.67±10.58	52.00±16.11	1.008	0.329
Risk factors, n (%)				
Hypertension	4 (33.33)	3 (50.00)		0.627^a^
Diabetes	1 (8.33)	1 (16.67)		1.000^a^
Hyperlipidemia	2 (16.67)	1 (16.67)		1.000^a^
Smoking	2 (16.67)	0 (0.00)		0.529^a^
Other data, mean±SD				
Systolic pressure (mmHg)	131.00±10.60	135.60±18.61	0.519	0.626
Diastolic pressure (mmHg)	80.33±5.19	89.80±19.83	1.053	0.349
Triglycerides (mmol/L)	1.31±0.70	1.08±0.28	0.711	0.491
Total cholesterol (mmol/L)	4.07±0.69	3.62±0.78	1.103	0.292
HDL-C (mmol/L)	1.18±0.25	1.22±0.11	0.404	0.694
LDL-C (mmol/L)	2.40±0.52	1.94±0.69	1.396	0.188
Glucose (mmol/L)	4.74±0.61	5.43±1.83	0.818	0.456
WMH volume (mL)	0.39±0.36	22.85±12.99	4.233	0.008
CADASIL: cerebral autosomal dominant arteriopathy with subcortical infarcts and leukoencephalopathy; HDL-C: high-density lipoprotein cholesterol; LDL-C: low-density lipoprotein cholesterol; WMH: white matter hyperintensity. ^a^Fisher exact test.				

### Differences in cerebral blood flow between the control and CADASIL groups

**Figure 1:**
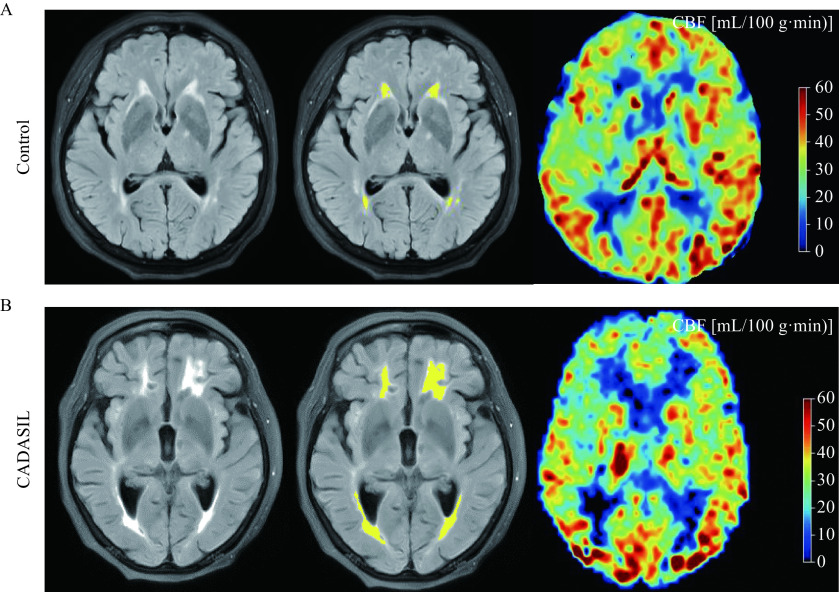
Maps of cerebral blood flow in the control and CADASIL groups.

Herein, we estimated mean CBF values of all participants, and ***Fig. 1*** shows representative maps of CBF in the control and CADASIL groups. CBF in WMH region was significantly lower in the CADASIL group [(23.31±2.39) mL/(100 g·min) than that in the control group [(33.64±1.19) mL/(100 g·min)](*t*=9.982, *P*<0.001). CBF in NAWM region was significantly low in the CADASIL group [(32.27±1.46) mL/(100 g·min) compared to the control group [(37.04±1.28) mL/(100 g·min)] (*t*=7.138, *P*<0.001). CBF in GM region was significantly reduced in the CADASIL group [(36.18±1.23) mL/(100 g·min)] compared to the control group [(39.84±1.47) mL/(100 g·min)] (*t*=5.244, *P*<0.001). Generally, the global CBF was also significantly lower in the CADASIL group [(29.08±2.34) mL/(100 g·min)] than that in the control group [(39.02±1.23) mL/(100 g·min)] (*t*=11.967, *P*<0.001) (***Table 2***).

**Table 2:** Differences in cerebral blood flow between the control and CADASIL groups

Regions	Control [mL/(100 g·min)]	CADASIL [mL/(100 g·min)]	*t*	*P*
WMH	33.64±1.19	23.31±2.39	9.982	<0.001
NAWM	37.04±1.28	32.27±1.46	7.138	<0.001
GM	39.84±1.47	36.18±1.23	5.244	<0.001
Global	39.02±1.23	29.08±2.34	11.967	<0.001
CBF: cerebral blood flow; CADASIL: cerebral autosomal dominant arteriopathy with subcortical infarcts and leukoencephalopathy; WMH: white matter hyperintensity; NAWM: normal-appearing white matter; GM: gray matter.				

### The correlation between cerebral blood flow and white matter hyperintensity volume

The CBF in the following regions of the brain was negatively correlated with the WMH volume: WMH (*r*=−0.710, *P*=0.001), NAWM (*r*=−0.586, *P*=0.011), GM (*r*=−0.545, *P*=0.019), and global brain (*r*=−0.748, *P*<0.001).

### The association between cerebral blood flow and white matter hyperintensity volume

Spearman's rank correlation test showed that the CBF in the following brain regions was positively related to WMH CBF: NAWM (*r=*0.657, *P=*0.003), GM (*r=*0.606, *P=*0.008), and global brain (*r=*0.647, *P=*0.004). After including CBF of different brain regions as multiple independent variables, we found that VIF was 10.17 (>10). We further corrected WMH CBF and analyzed the association of CBF with the WMH volume in other brain regions.

It was found that an increase of 1 mL/(100 g·min) in the CBF in WMH was associated with a WMH volume reduction of 1.77 mL (*P=*0.001). After adjusting for the potential confounding factors (gender, age, hypertension, diabetes mellitus, dyslipidemia, and smoking), the WMH volume was found to decrease by 2.23 mL for each 1 mL/(100 g·min) increase in WMH CBF (*P*<0.001). After adjusting for WMH CBF and other potential confounding factors (gender, age, hypertension, diabetes mellitus, dyslipidemia, and smoking), the association between CBF in other brain regions and the WMH volume was not found to be statistically significant (*P*>0.05, ***Table 3***).

**Table 3:** The association between cerebral blood flow and white matter hyperintensity volume

CBF	Crude *β* (95% CI)	*P*	Adjusted *β* (95% CI)	*P* ^a^
WMH CBF	−1.77 (−2.68, −0.86)	0.001	−2.23 (−3.04, −1.41)	<0.001^a^
NAWM CBF	−3.43 (−5.28, −15.78)	0.001	−0.23 (−3.51, 3.04)	0.875^b^
GM CBF	−3.46 (−5.93, −0.99)	0.009	0.12 (−3.24, 3.49)	0.935^b^
Global CBF	−2.04 (−2.85, −1.22)	<0.001	−0.23 (−2.85, 2.39)	0.846^b^
^a^Adjusted for age, gender, hypertension, diabetes mellitus, dyslipidemia, and smoking; ^b^adjusted for age, gender, hypertension, diabetes mellitus, dyslipidemia, smoking, and WMH CBF. CI: cofidence interval; CBF: cerebral blood flow; WMH: white matter hyperintensity; NAWM: normal-appearing white matter; GM: gray matter.				

## Discussion

CADASIL is a common genetic SVD that presents with typical pathological changes of Notch3^ECD^ deposition and small vessel degeneration^[23]^. These marked degenerative changes lead to cerebrovascular dysfunction, which further causes white matter tract damage^[24]^. Interestingly, mutant NOTCH3 occurs in both the brain and peripheral arterial vessels^[8]^. Conversely, peripheral arteriopathy is subclinical, and small arterial function is not impaired^[8]^. In addition, the white matter region is most vulnerable to cerebrovascular dysfunction in CADASIL. The mechanism of its increased vulnerability remains unknown. Thus, in this study, we divided the brain into several regions and observed the cerebrovascular function changes individually in these regions to explore whether compromised cerebrovascular function only occurred in the WMH region. Using ASL-MRI, we analyzed the CBF in WMH, NAWM, GM, and global brain in the CADASIL group. We also investigated the effects of the cerebral perfusion status in different regions on WMH. Our findings revealed that the WMH volume was significantly increased in the CADASIL group compared with that in the control group. CBF in all brain regions was significantly decreased in the CADASIL group. CBF reduction in WMH region was positively related to CBF in NAWM, GM, and global brain regions. WMH severity was only associated with CBF in WMH region but not in NAWM, GM, and global regions.

In CADASIL, *NOTCH3* expression is predominant in VSMCs and pericytes of the arteries, arterioles, and capillaries of small vessels^[3]^. VSMCs and pericytes are mural cells wrapping around small vessels; they have contractile properties and participate in CBF regulation^[13]^. Thus, *NOTCH3* mutations predominantly affect the cerebral perfusion function of small cerebral vessels in CADASIL. In addition, in the early stages of CADASIL, MRI lesions are mostly detected in the deep WM^[25]^. We first examined the CBF changes in WMH region and found it lower in CADASIL compared with the controls. A previous study also reported significantly reduced CBF in WMH, and this reduction was more severe in patients with clinical cognitive impairment^[26]^, which was consistent with our study.

Furthermore, pathological studies have demonstrated that all the small cerebral vessels are involved in the deposition of GOM^[7]^. GOM deposition around the cell surface causes structural damage to brain micro vessels, including VSMC and pericyte degeneration, increased basement membrane thickness, and vessel wall destruction^[5]^. These microstructural changes in the brain may also contribute to CBF reduction^[27]^. However, it remains largely unknown whether the neurovascular functions of other brain regions besides WMH are compromised as well. In the current study, we found significantly reduced CBF in all four regions. A single-photon emission computerized tomography study of CADASIL patients also reported CBF reduction in the basal ganglia^[28]^. This indicates that cerebral hypoperfusion is global and not region-specific and that cerebrovascular function is overall deteriorated in patients with CADASIL, which is consistent with the vascular degeneration pattern.

We further analyzed the relationship between CBF of different brain regions. Notably, CBF in NAWM, GM and global regions was positively correlated with CBF in WMH region. This indicates CBF reduction in WMH region occurs in the early stage of CADASIL, followed by in NAWM and GM regions. One possible reason could be that nerves and vessels in WMH areas are more vulnerable to GOM deposition. At the early stage of WMH formation, CBF in NAWM and GM may retain. With the further aggravation of small vessel function in WMH region, neurovascular function in the GM region is also compromised, leading to global hypoperfusion. After correction analysis, only CBF in WMH region was associated with WMH volume, further indicating the contribution of small vessel function in WMH region in the pathological changes of CADASIL. Notably, decreased CBF supply in the WMH region occurred in the early stages of CADASIL^[10]^. This also corroborates reports of other clinical studies that neurovascular dysfunction was the early pathological change in patients with CADASIL; neurovascular dysfunction is often detected concurrently to lowered CVR^[16,29]^, which is another useful predictor of WMH burden. Furthermore, the reduced velocity of lenticulostriate arteries precedes the formation of lesions identified in the MRI scans^[17]^. These results suggest that CBF in WMH region can serve as a candidate imaging indicator for monitoring the WMH volume in CADASIL.

Our study had several limitations. First, the number of participants is limited and these patients may not fully represent the characteristics of CADASIL patients. Further larger and multicenter cohort studies are needed to confirm the results. Second, it is a cross-section study. The prospective imaging data and clinical status during the follow-up observation need to be included in further study. Whether a better understanding of the WMH region can help us diagnose and predict the prognosis of CADASIL needs to be explored. In addition, although NVU dysfunction precedes WMH formation and is the primary cause of WMH, the regulatory mechanisms of NVU dysfunction in CADASIL are largely unknown. Currently, most researches are concentrated on GOM-mediated VSMC dysfunction. We propose that other mediators are also involved in the pathology of NVU dysfunction in CADASIL, such as astrocytes. This hypothesis needs to be tested in subsequent studies.

CADASIL is a monogenic disease caused by *NOTCH3* mutations. Compromised NVU function is significantly associated with the initiation and progression of WMH in CADASIL. CBF decreased in all different brain regions, and lower CBF in WMH region is associated with a higher WMH volume in CADASIL, suggesting the overall deterioration of NVU function in CADASIL. Monitoring NVU function in specific brain regions may be a useful approach to identify an early time point for preemptive intervention in CADASIL.
